# Paediatric emergency care at an academic referral hospital in Mozambique

**DOI:** 10.1016/j.afjem.2021.07.003

**Published:** 2021-10-14

**Authors:** Hajra Ismail, Harshika Chowdhary, Breena R. Taira, Solange Moiane, Laila Faruk, Benilde Alface, Jyodi Mohole, Otília Gonçalves, Emily A. Hartford, W. Chris Buck

**Affiliations:** a Hospital Central de Maputo, Maputo, Mozambique; b University of California Los Angeles, David Geffen School of Medicine, Los Angeles, USA; c Olive View-UCLA Medical Center, Sylmar, CA, USA; d University of Washington Department of Pediatrics, Emergency Medicine, Seattle, WA, USA

**Keywords:** Paediatric, Emergency care, Sub-Saharan Africa, Triage

## Abstract

**Background:**

Improved emergency care of children with acute illness or injuries is needed for countries in Africa to continue to reduce childhood mortality rates. Quality improvement efforts will depend on robust baseline data, but little has been published on the breadth and severity of paediatric illness seen in Mozambique.

**Methods:**

This was a retrospective review of routinely collected provider shift summary data from the Paediatric Emergency Department (PED) at Hospital Central de Maputo (HCM), the principal academic and referral hospital in the country. All children 0–14 years of age seen in the 12-month period from August 2018–July 2019 were included. Descriptive statistical analyses were performed.

**Results:**

Data from 346 days and 64,966 patient encounters were analyzed. The large majority of patients (96.4%) presented directly to the PED without referral from a lower level facility. An average of 188 patients was seen per day, with significant seasonal variation peaking in March (292 patients/day). The most common diagnoses were upper respiratory infections (URI), gastroenteritis, asthma, and dermatologic problems. The highest acuity diagnoses were neurologic problems (59%), asthma (57%), and neonatal diagnoses (50%). Diagnoses with the largest proportion of admissions included neurologic problems, malaria, and neonatal diagnoses. Rapid malaria antigen tests were the most commonly ordered laboratory test across all diagnostic categories; full blood count (FBC) and chemistries were also commonly ordered. Urinalysis and HIV testing were rarely done in the PED.

**Conclusion:**

This epidemiologic profile of illness seen in the HCM PED will allow for improved resource utilisation. We identified opportunities for evidence-based care algorithms for common diagnoses such as respiratory illness to improve patient care and flow. The PED may also be able to optimize laboratory and radiology evaluation for patients and develop standardized admission criteria by diagnosis.

## African relevance

• Reducing child mortality is still a priority for many African countries and provision of paediatric emergency care is crucial
• There is little data on the epidemiology, disease burden, and resource utilisation of paediatric emergency care across Africa
• This study analyzes data from a large academic referral hospital in Mozambique and informs priorities and future work

## Introduction

Over the past two decades childhood mortality has decreased globally, but gains differ across geographical regions. Between 1990 and 2018, the under-five mortality rate decreased from 93 deaths (per 1000 live births) to 39 worldwide. However, in Africa, the under-five mortality rate remains high at 76 deaths per 1000 with great variation across the continent [1].

In Mozambique, childhood mortality improved during this timeframe with under-five rates decreasing from 240 to 73 per 1000, and infant (<12 month) mortality rates decreasing from 161 to 54, but has not yet reached the Sustainable Development Goal of 25 deaths per 1000 live births and remains in the bottom 15% globally 1., 2.. While continued expansion of general child health programs such as the Expanded Program on Immunization and Integrated Management of Childhood Illness continue to be key pillars for further reduction of paediatric mortality, there is growing recognition of the importance of improved emergency care for children who present to health facilities with acute illness or injuries 3., [4].

Programs such as the World Health Organization's Emergency Triage Assessment and Treatment (ETAT) have been shown to decrease mortality after being implemented in sub-Saharan African paediatric emergency care centers, as the majority of in-hospital mortality occurs within the first 48 h after presentation [5], [6]. Increased attention to Paediatric Emergency Medicine as a specialty with corresponding development of care algorithms, curricula, and training programs have also been shown to either positively impact mortality or have been proposed as means to improvement 3., 7., 8., 9., [10]. Emergency medicine in Mozambique is also newly developing as a specialty, but little has been published regarding paediatric emergency care in the country.

Data is needed to inform ongoing efforts to reduce overall inpatient mortality and establish a baseline for quality improvement initiatives. The goal of this study is to describe the volume, breadth, and severity of illness among patients presenting to the Hospital Central de Maputo (HCM) Paediatric Emergency Department (PED) over the course of one year.

## Methods

This was a retrospective, descriptive study evaluating routinely collected departmental statistics summarizing the patients seen each day in the PED at HCM.

Inclusion criteria were all patients 0–14 years of age registered in the PED from August 1, 2018 to July 31, 2019. Patients ≥15 years of age are generally referred to the adult emergency center and were not included in this analysis.

HCM is located in Maputo, the capital city of Mozambique, a country with a population of approximately 30 million people. It is the principal referral and academic hospital for the country. There are other two other hospitals with paediatric specialists within Maputo City, but HCM has the highest level of intensive care and subspecialist availability. HCM receives patients transferred from health centers for emergency center evaluation or admission, from other hospitals for subspecialty care, as well as presenting directly from home. The PED at HCM has approximately 69,000 annual visits. ETAT was implemented in 2013. It is staffed primarily by general medical doctors and paediatric nurses, with support from paediatric residents and specialists. It serves as a training site for medical students, nursing students, and residents. Per 8-hour shift, there are usually five general medical doctors, one resident, two nurses, two lab assistants, and two medical assistants focused on initial registration and triage. The PED includes a waiting room, a triage room, four consultation rooms, and treatment area with five beds. The treatment area is used to administer nebulizers (salbutamol, epinephrine, and saline), intravenous (IV) fluids, and medications. In the PED, ampicillin, gentamicin, ceftriaxone, metronidazole, penicillin, and artesunate are routinely available antimicrobials. Medications such as prednisolone and hydrocortisone are also available in oral, intramuscular, and intravenous formulations. There is no electronic medical record system in the PED.

The PED has a co-located pharmacy, laboratory, and basic radiology suite. Abdominal and thoracic x-rays are completed on a limited basis. Malaria tests, complete blood counts, urinalysis, biochemistry panels, and HIV tests are completed in the PED lab, additional labs are sent to the hospital's central laboratory. CT scans and ultrasounds are not routinely available. The PED is located immediately adjacent to the paediatric intensive care unit (PICU), and critically ill patients, including those with major trauma, may pass directly through to the PICU for resuscitation. Therefore, deaths occurring prior to or shortly after arrival to the PED are recorded in the PICU, where the mortality rate has been reported as 25% [11].

As the PED does not have an electronic medical record or an encounter database that uses ICD10 diagnosis codes, the data source for the study were shift summary sheets completed by medical doctors at the end of each shift. These sheets include demographic information (age and sex), referral source, triage level (A-very urgent, B-urgent, C-not urgent, D-priority/child of healthcare worker), diagnostic testing ordered, diagnoses, and disposition. The data source used for this study does not contain information on patient deaths. Individual patient charts/medical records were not used. It was not feasible to create a patient-level database for this study, so investigators manually aggregated provider shift summary sheets into a daily departmental summary table, created for this study, containing data for all providers who worked each day, organized by diagnostic categories that align with standard departmental reporting. The data from these daily summary tables was then entered into a RedCap database [12].

Descriptive statistics were used to summarize demographic characteristics, referral source, triage levels, diagnostic tests ordered, and PED disposition. Based on the format of these daily summary tables, analysis focused on visit characteristics according to main diagnostic categories, but analysis between other variables was not possible. Results were disaggregated by month to evaluate temporal trends over the course of the 12-month study period. All statistical analysis was performed using STATA®.

The study protocol was approved by Scientific Directorate of HCM, the Institutional Review Board (IRB) of the University Eduardo Mondlane School of Medicine and HCM (CIBS FM&HCM – 825881101), and the University of California Los Angeles IRB (#13-000579-AM-00004). Informed consent was not required.

## Results

Data were available for 346 days of patient care from August 1, 2018 to July 31, 2019. There were 19 missing days of data: seven from June, four from January, two from July, August and September, one from May and one from December. A total of 64,966 patients were seen in the PED in this time period. Of these patients, 96.4% presented directly without having been referred from a primary healthcare facility.

**Fig. 1:**
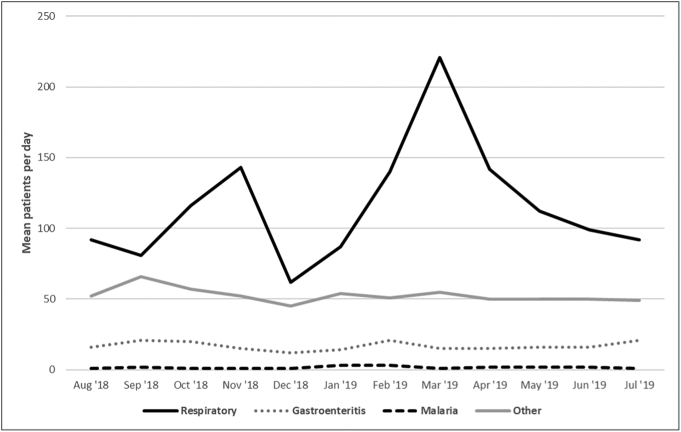
Annual distribution of mean patients per day by diagnosis. Note: Respiratory line includes asthma, bronchiolitis, pneumonia, and upper respiratory tract infections diagnostic categories. Other line includes dermatologic, trauma, other surgical, neurologic, neonatal, and other diagnostic categories.

The daily average number of patients seen was 188 over the course of the 12-month study period. There was seasonal variation with the highest mean number of patients seen per day in March (292/day) and the lowest in December (120/day). This variation was largely driven by the volume of respiratory illness, while average daily patient visits for malaria and gastroenteritis were relatively stable over the course of the year (Fig. 1).

The age distribution for patients was 2.2% aged 0–28 days, 18.1% aged 29 days-12 months, 45.9% ages 1–4 years, 22.8% ages 5–9 years, and 11.1% age 10–14 years. Upper respiratory infections (URI, mean 84/day), gastroenteritis (mean 17/day), asthma (mean 15/day), and dermatologic illness (mean 14/day) were the most frequent diagnoses, and malaria the least frequent (mean 2/day) across all age groups. URIs were responsible for the most visits per day in all age groups except patients 0–28 days. The percentage of daily visits due to asthma increased with increasing patient age, with a maximum of 14% of visits in the 10–14 year age group. Children under five years (except for neonates) had the highest percentage of daily visits due to gastroenteritis. Infants had the most daily visits for dermatologic problems. Further breakdown of daily visits by age and diagnosis are provided in Table 1.

**Table 1:** Mean visits per day by diagnosis, disaggregated by age.

Diagnosis	0–28 days	1 month–12 months	1 year–4 years	5 years–9 years	10 years–14 years	Total
Asthma	0 (0%)	0.7 (2%)	6.3 (7%)	4.7 (11%)	3 (14%)	14.7 (8%)
Bronchiolitis	0 (0%)	3 (9%)	0.2 (0%)	0 (0%)	0 (0%)	3.2 (2%)
Pneumonia	0 (0%)	2 (6%)	7.4 (9%)	3 (7%)	1.5 (7%)	13.9 (7%)
URI	0.6 (15%)	14 (43%)	41.6 (48%)	19.3 (46%)	7.5 (36%)	83.0 (45%)
Gastroenteritis	0.1 (2%)	4.1 (2%)	8.4 (10%)	3.1 (7%)	1.3 (6%)	17.0 (9%)
Malaria	0 (0%)	0.1 (0%)	0.6 (1%)	0.5 (1%)	0.4 (2%)	1.6 (1%)
Dermatologic	0.2 (5%)	3.2 (10%)	6.7 (8%)	3.1 (7%)	1.1 (5%)	14.3 (8%)
Trauma	0 (0%)	0.2 (1%)	1.1 (1%)	0.8 (2%)	0.5 (2%)	2.6 (1%)
Other surgical	0.1 (2%)	0.8 (2%)	1.3 (2%)	0.8 (2%)	0.6 (3%)	3.6 (2%)
Neurologic	0.1 (2%)	0.4 (1%)	0.9 (1%)	0.4 (1%)	0.4 (2%)	2.2 (1%)
Neonatal	2.6 (63%)	0 (0%)	0 (0%)	0 (0%)	0 (0%)	2.6 (1%)
Other	0.4 (10%)	4.4 (13%)	11.3 (13%)	6.6 (16%)	4.4 (21%)	27.1 (15%)
Total mean visits per day	4.1 (100%)	32.9 (100%)	85.8 (100%)	42.3 (100%)	20.7 (100%)	185.8 (100%)

URI, upper respiratory infection.

**Fig. 2:**
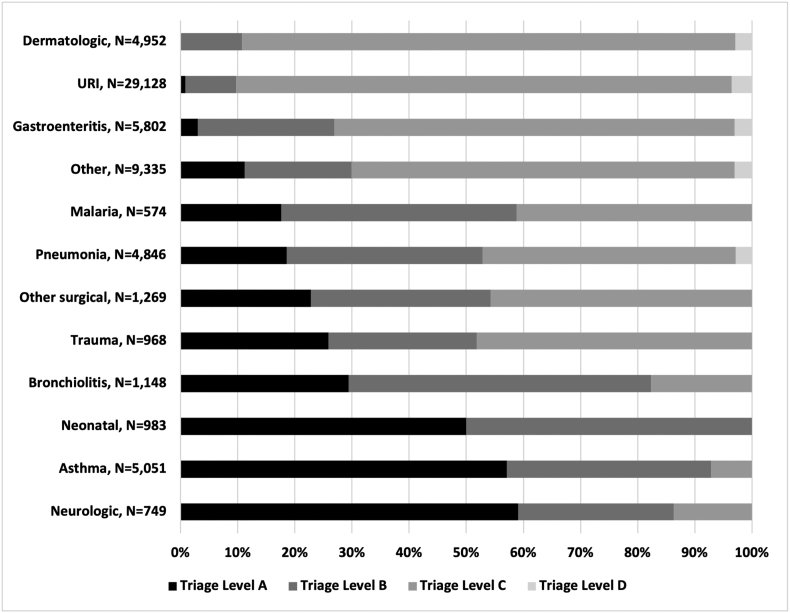
Triage level by diagnosis, all age groups, aggregate annual data. Note: URI = upper respiratory infection.

Diagnoses that were more commonly classified as “very urgent” (ETAT A) included neurologic diagnoses, asthma, and neonatal diagnoses at 59%, 57%, and 50%, respectively. URIs and dermatologic diagnoses were more commonly classified as lower acuity, 87% and 86% were non-urgent triage classification C, respectively. Further details by diagnosis are provided in Fig. 2.

Rapid malaria tests were the most commonly ordered test across all diagnoses. Both full blood counts (FBC) and chemistry panels were most commonly ordered in patients with malaria and neurologic diagnoses. Urinalysis and HIV rapid tests were infrequently ordered relative to other laboratory tests. Chest radiographs (CXR) were obtained in 54% of pneumonia, 36% of bronchiolitis, 19% of asthma, and 2% of upper respiratory infection diagnoses. Further details of diagnostic testing performed by diagnosis is presented in Table 2.

**Table 2:** Ratio of mean number of laboratory tests and chest radiographs ordered per day to mean number of visits per day by diagnosis, aggregate annual data.

	Malaria	FBC	UA	Chem	HIV	CXR
Asthma	29%	23%	<1%	3%	1%	19%
Bronchiolitis	58%	51%	3%	15%	3%	36%
Pneumonia	62%	52%	2%	7%	2%	54%
URI	51%	25%	<1%	<1%	<1%	2%
Gastroenteritis	54%	39%	2%	6%	2%	3%
Malaria	88%	88%	29%	47%	11%	2%
Dermatologic	24%	16%	<1%	3%	1%	1%
Trauma	18%	18%	4%	11%	7%	29%
Other surgical	46%	43%	14%	27%	14%	30%
Neurologic	73%	68%	23%	45%	23%	23%
Newborn	68%	64%	11%	50%	7%	11%
Other	44%	35%	9%	15%	6%	11%

FBC, full blood count; UA, urinalysis; chem, biochemistry panel; HIV, human immune deficiency virus rapid test; CXR, chest radiograph.

**Fig. 3:**
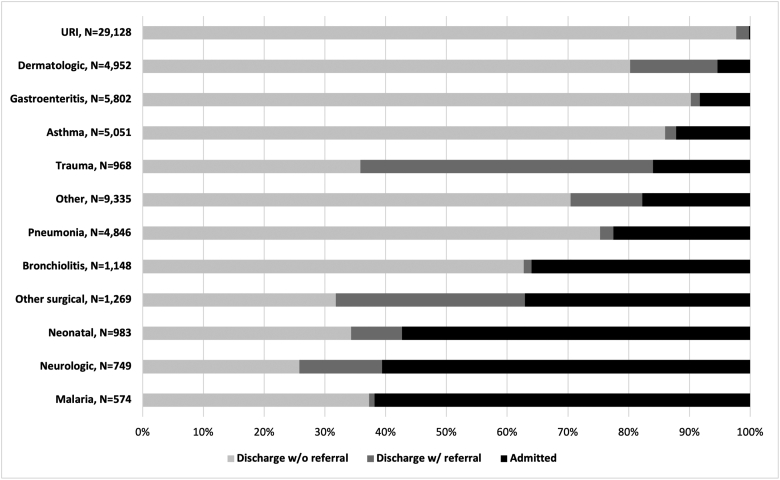
Disposition, all age groups, aggregate annual data.

The diagnoses most frequently admitted to the hospital were malaria, neurologic diagnoses, and neonatal diagnoses at 62%, 61%, and 57%, respectively. For the respiratory categories, admission rates were 36% for bronchiolitis, 23% for pneumonia, 12% for asthma, and <1% for URIs. Of the 88% of asthma patients not admitted to the hospital, only 2% received a referral for follow-up outpatient care. Further information on disposition by diagnosis is provided in Fig. 3.

## Discussion

This is an analysis of one year of data from the PED at HCM, the principal referral and academic center in Mozambique. There were a high number of patient encounters with a total of approximately 65,000 for the year. Patient volumes peaked in March at 292/day corresponding with the rainy season and peak in respiratory illness and were the lowest in December and during the dry season months in July and August. Interestingly, despite the designation of HCM as a referral hospital, the vast majority of patients presented from home despite higher out of pocket costs for patients presenting to the PED without referral. This may reflect patient and family perceptions of higher quality care available at HCM. A recent analysis of emergency center visits from Hospital Central de Beira (HCB), the referral hospital for central Mozambique, found that a much larger proportion of patients (44%) were referred from primary health centers or lower level hospitals [13].

Overall, 62% of patients had primary respiratory complaints, compared to only 29% reported from HCB [13]. This is likely due to HCM seeing many more patients presenting directly from home without referral, as 73% of all respiratory diagnoses were URIs, and across all age groups, this was the most common diagnosis. The majority of these were classified as lower acuity, although approximately 10% were urgent indicating a more severe illness. Pneumonia, bronchiolitis, and asthma were also commonly seen. It can be difficult to differentiate between aetiologies of paediatric respiratory illness, but the age groupings for bronchiolitis mostly under age one year and asthma becoming more frequent with increasing age suggest appropriate diagnostic categorization.

A high amount of resources were used for patients with respiratory illness. Bronchiolitis is a common illness in infancy, and it is a clinical diagnosis that does not depend on laboratory or radiographic testing. However, the majority of patients with bronchiolitis received a malaria test and FBC, and approximately 1/3 had a CXR. These findings suggest that the diagnosis and management of bronchiolitis may be an area for improved use of resources in our setting. Historically, approximately 2–3% of primary bronchiolitis infections result in admission [14], [15]. There is variation around the world as to the likelihood of admission after presentation to a PED. Recent trends in England reported a median admission proportion of 4.6% whereas in the United States there is a large amount of variation in admission rates by hospital and region between 19 and 65% [16], [17]. The wide variation may be due to practice differences or differences in patient severity at presentation. In our study, 35% of patients with bronchiolitis were admitted to the hospital, and a large proportion received an urgent triage designation indicating that the initial acuity was high. Despite many trials of treatments for bronchiolitis, only supportive care with hydration, oxygen, and management of secretions has been proven effective and thus criteria for admission in any setting should be related to the need for supportive care [18].

The majority of patients with asthma were assigned an urgent triage level. 19% received a CXR and 23% had an FBC. Often patients with known asthma presenting with an exacerbation do not require imaging or labs. The majority were treated in the PED and discharged with only 12% requiring admission to the hospital. However, it was notable that 86% patients were discharged with a referral follow up. This is likely in part due to the limited capacity for outpatient follow-up of chronic illness related to scarcity of physician human resources, but still is a possible area for improvement, as routine outpatient care for asthma is important for prevention of exacerbations.

Pneumonia was another common diagnosis, most commonly seen in the 1–4 year age range where it is known to be a significant cause of mortality [2]. About half of patients with pneumonia received a CXR and FBC. There is debate in the international literature about definitive diagnosis of pneumonia and if it is purely a clinical diagnosis or requires imaging [19]. The World Health Organization (WHO) and IMCI use fast breathing and/or chest indrawing as evidence of pneumonia without the requirement for imaging [20]. These guidelines promoting a non-specific clinical diagnosis of pneumonia may be more applicable in facilities with fewer resources than our PED. Ultrasound is an emerging modality with high sensitivity and specificity for the diagnosis of paediatric pneumonia and may be useful in the HCM PED in the future 21., 22.. The majority of patients with pneumonia were discharged which reflect management in concordance with international guidelines for outpatient management unless a sign of severe illness is present [20].

Gastroenteritis and dermatologic complaints were also commonly seen across age groups but most commonly in younger children. For those with gastroenteritis, a significant portion (30%) presented with an urgent triage level, and the majority received laboratory testing, only 10% were admitted. Our PED does not have access to ondansetron, an antiemetic which is on the WHO essential medicines list, and has been shown to help reduce the need for admission in children with gastroenteritis with vomiting 23., [24]. Mozambique introduced rotavirus vaccine into the national immunization schedule in 2015, with reduction in the number of admissions for diarrheal disease [25].

Malaria was seen throughout the year in the HCM PED but with relatively low frequency. Unlike other parts of the country, Maputo City and Maputo Province in the southernmost part of Mozambique have low malaria prevalence (parasitemia in less than 3% of children randomly selected for testing) [26]. This low prevalence combined with strong capacity to diagnose and treat uncomplicated malaria at primary health centers can help explain the relatively small proportion of malaria cases seen in this review. Many of the malaria patients seen in the HCM PED are transfers from other hospitals with severe malaria, requiring hospitalization.

There were tests infrequently utilised in the PED that likely represent an area for improvement. Very few patients received a urinalysis and thus urinary tract infection was virtually absent as a diagnosis. Urine samples are difficult to obtain in paediatric patients, particularly those who are not toilet trained and require catheterization. However, UTI is a common bacterial infection in paediatric patients and lack of routine testing for infants with fever likely represents a significant amount of missed UTIs. Timely urinalysis testing will be an important focus going forward, and non-invasive collection methods can be considered [27].

Maputo City and Maputo Province had estimated adult HIV prevalence of 16.9% and 22.9% in 2015, and in that context, the frequency of HIV testing in the PED may seem low. Mozambique guidelines for provider-initiated HIV testing and counseling (PITC) recommend sign and symptom-based testing for outpatients and routine testing for inpatients 28., 29.. During the time period of this study, patients being admitted often had their HIV testing performed on the wards and we were not able to capture that data. More recently, a counselor has been allocated to the PED so that timely testing of children or breastfeeding mothers can occur at the initial point of contact, and future analyses will be better able to measure PITC rates in the PED.

The diagnoses with the highest acuity were neurologic, asthma, and neonatal problems. Neurologic diagnoses included seizures, altered mental status, meningitis or meningoencephalitis. The PED receives a mean of two patients per day with a neurologic diagnosis. Protocols, training, and simulation to quickly assess and treat patients with these diagnoses may be helpful given they are less common than other diagnoses but still likely to be seen on a daily basis. Neonates are grouped together under one diagnosis, so it is not possible to analyze their specific diagnoses here. The high acuity is likely due to those with sepsis, severe dehydration, or jaundice, and ETAT guidelines call for sick neonates less than two months of age to receive priority attention [30].

Our study was a retrospective review of one year of patient data. Despite every effort to locate the data, we had 19 days of missing data. It is unlikely that this meaningfully altered our results since, with the exception of June (which is a lower volume month), they were scattered over the course of the year. The principal limitation in analysis was that our data source was shift summary sheets filled out by PED physicians, and not individual patient records. Study investigators used these sheets to compile a daily summary sheet to categorize diagnoses in alignment with standard departmental reporting whereby more frequent respiratory illnesses are disaggregated, but other less commonly seen diagnosis are grouped by system. A standardized approach was used for classification of diagnoses into categories, but there may have been some inconsistencies between investigators. The daily summary sheets were organized by a single variable (diagnostic category was chosen) which meant that analysis of all variables by diagnostic category was possible, but we were not able to look at the relationships between other variables (i.e. triage and age). There was no treatment data available, and we were not able to include a meaningful analysis of mortality as critically ill patients are admitted directly into intensive care services.

## Conclusions

In this baseline study of paediatric disease in the HCM PED, there were many interesting findings to inform future work and quality improvement efforts. The majority of patients presented from home despite this being a referral center. The most common diagnoses were respiratory, and we identified opportunities for improved resource utilisation including laboratory testing and CXR and standardized admission criteria for these diagnoses. We also identified lack of urinalysis and HIV testing. Future research can focus on the implementation of evidence-based care algorithms for areas where gaps have been identified.

## Authorship contribution statement

Authors contributed as follow to the conception or design of the work; the acquisition, analysis, or interpretation of data for the work; and drafting the work or revising it critically for important intellectual content: HI and HC each contributed 25%; EH and CB contributed 10% each; BT, SM, LF, BA, JM, and OG all contributed 5%. All authors approved the version to be published and agreed to be accountable for all aspects of the work.

## Declaration of competing interest

The authors declare no conflicts of interest.
